# Potential utility of amantadine DR/ER in persons with Parkinson’s disease meeting 5-2-1 criteria for device aided therapy

**DOI:** 10.1016/j.prdoa.2021.100123

**Published:** 2021-12-08

**Authors:** Robert A. Hauser, Santosh Goud, Andrea E. Formella

**Affiliations:** a University of South Florida, Tampa, FL, USA; b Adamas Pharmaceuticals, Inc., Emeryville, CA, USA

**Keywords:** Amantadine, 5-2-1 criteria, Parkinson’s disease, Dyskinesia, OFF time, ON time, Treatment

## Abstract

**Background:**

The 5-2-1 criteria (≥5 levodopa doses/day, ≥2 h OFF/day, and ≥ 1-hour dyskinesia/day) propose to identify people with Parkinson’s disease (PD) who are poorly controlled on oral therapies and who may therefore benefit from device-aided therapies. Amantadine-DR/ER is the only medication FDA-approved for both dyskinesia and OFF episodes in levodopa-treated patients. In this *post-hoc* analysis of phase 3 clinical trials, we evaluated the efficacy and safety of amantadine-DR/ER in patients meeting 5-2-1 criteria.

**Methods:**

Week-12 treatment differences (Amantadine-DR/ER − placebo) in the Unified Dyskinesia Rating Scale (UDysRS) and PD motor states (patient diaries) were evaluated in pooled, phase-3, double-blind trial participants meeting 5-2-1 criteria at baseline. This 5-2-1 cohort was followed into a 2-year open-label trial, where Movement Disorder Society - Unified Parkinson’s Disease Rate Scale (MDS-UPDRS) Part IV scores were assessed relative to double-blind baseline.

**Results:**

Of 198 enrolled participants in the phase 3 trials, 65 (33%; n = 29 placebo; n = 36 amantadine-DR/ER) comprised the 5-2-1 cohort. At Week-12 endpoint, amantadine-DR/ER significantly improved UDysRS scores (treatment difference of 9.57 ± 3.15 points, p = 0.004) and ON time without troublesome dyskinesia (‘good ON’, treatment difference of 2.9 ± 0.90 h/day, p = 0.002). Improvements in good ON time resulted from significant reductions in both troublesome dyskinesia and OFF time. Treatment benefit on MDS-UPDRS-Part IV was sustained through open-label, follow-up. The most common adverse events in patients who met 5-2-1 criteria and were treated with amantadine-DR/ER included falls and peripheral edema.

**Conclusions:**

Findings suggest Amantadine-DR/ER should be considered as an option for people with PD who meet 5-2-1 criteria.

## Highlights

• 5-2-1 criteria help identify PD patients who may benefit from device aided therapy.
• Amantadine-DR/ER increased ‘good’ ON time in patients meeting 5-2-1 criteria.
• Increased good ON time resulted from reductions in troublesome dyskinesia and OFF.
• Amantadine-DR/ER efficacy was maintained over 100 weeks in this advanced population.

## 1 Introduction

Advanced PD is often characterized by poor control of motor features despite optimization of oral and transdermal dopaminergic agents, often leaving patients to manage living with dyskinesia and being OFF or frozen for as much as 50% of their waking day [1]. At this stage, many clinicians will consider device aided therapies (DAT) such as subcutaneous-apomorphine infusion (SCAI), levodopa/carbidopa intestinal gel (LCIG) infusion or deep brain stimulation (DBS) for their patients.

The 5-2-1 criteria (≥5 levodopa doses, ≥2 h OFF, and ≥ 1-hour dyskinesia per day) were proposed as a simple means of identifying patients with ‘advanced’ disease who are uncontrolled on oral/transdermal therapies and may therefore benefit from DATs [2]. By the time patients meet these eligibility criteria for DAT, they can be on multiple medications, including a variety of dopamine agonists, MAO-B inhibitors and COMT inhibitors in addition to their levodopa [3]. However, observational studies conducted at specialist PD centers show that only around a third (27–33%) of patients are on amantadine before referral for DAT [4], [5], and database reviews suggest that the percentage of patients trying amantadine is even lower overall (∼10%) [6], [7], [8]. Reasons for this underuse have been suggested to include lack of robust evidence for the immediate-release formulations [9] as well as a lack of medical support on how to use this ‘old’ molecule in the context of modern PD therapy [10].

The bedtime-administered, amantadine delayed release/extended release (DR/ER) capsule formulation (ADS-5102; Gocovri®, Adamas Pharmaceuticals, Emeryville, CA) has recently received FDA-approval for both dyskinesia and/or OFF episodes in levodopa-treated patients inviting renewed attention to the potential of modified-release amantadine for patients with both types of motor complications. In phase 3 clinical trials, treatment with amantadine-DR/ER significantly reduced dyskinesia and OFF time [11], [12], [13] and these therapeutic effects persisted through a 2-year, open-label follow-on trial [14]. Patients recruited to the phase 3 studies had to be experiencing ≥ 1 h of ON time with troublesome dyskinesia per day, thus meeting one of the 5-2-1 criteria by virtue of eligibility criteria. However, a substantial proportion of patients were also receiving at least 5 levodopa doses per day and/or experiencing significant OFF time at baseline. In this analysis we sought to evaluate the efficacy of amantadine-DR/ER for motor complications in patients participating in the Amantadine DR/ER phase 3 studies who met 5-2-1 criteria for advanced PD, rendering them ostensibly eligible for DAT.

## 2 Methods

### 2.1 Trial designs and participants

We present *post-hoc* analyses from two pooled, phase 3, pivotal trials of Amantadine-DR/ER [11], [12] and the corresponding open-label follow-on trial [15]. All three trials were conducted in compliance with the Declaration of Helsinki and International Conference on Harmonization (ICH) Good Clinical Practice (GCP) guidelines; all participating sites received institutional review board approval and all participants provided written informed consent before any trial procedures. Full details of these trials have been previously published:

1. EASE LID: A randomized, double-blind, placebo-controlled, 25-week clinical trial (NCT02136914) conducted at 44 North American sites between May 2014 and July 2015 [11].
2. EASE LID 3: A randomized, double-blind, placebo-controlled, 12-week trial (NCT02274766) conducted at 39 sites in the US and Western Europe between October 2014 and December 2015 [12].
3. EASE LID 2: An open-label 2-year trial (NCT02202551), conducted between July 2014 and March 2016, and including participants from EASE-LID and EASE LID 3 [15].

Briefly, participants (aged 30–85 years old) in EASE LID and EASE LID 3 were required to be experiencing ≥ 1 h/day (2 half-hour intervals) of ON time with troublesome dyskinesia. Dyskinesia was required to be causing at least mild functional impairment, as documented at screening and baseline by a score ≥ 2 on item 4.2 of the Movement Disorder Society–Unified Parkinson’s Disease Rating Scale (MDS-UPDRS) [16]. Key exclusion criteria included atypical parkinsonism, any acute or major psychiatric disorder that would affect the subject’s ability to complete study assessments, neurosurgical intervention in PD, dyskinesia not caused by dopaminergic stimulation in PD, and use of amantadine within the previous 30 days.

Enrolled participants were randomized in a 1 : 1 ratio to double-blind amantadine-DR/ER or matching placebo administered as two capsules once daily at bedtime. Amantadine-DR/ER was initiated at 137 mg for the first week (administered as one capsule containing active drug, and one capsule containing placebo), and 274 mg (two 137-mg capsules) thereafter. For the two-capsule dose, the 274 mg amantadine content is equivalent to 340 mg of amantadine HCl. For the final week, the dose was reduced to 137 mg (170 mg amantadine HCl). Throughout the studies, each patient’s regimen of antiparkinsonian medications, including levodopa, remained unchanged.

Study participants completing these double-blind trials could continue into EASE LID 2 and receive open-label amantadine-DR/ER for up to 101 weeks, with or without a gap between double-blind and open-label trials [15]. Participants previously excluded from the pivotal trials due to use of a deep brain stimulation device and those who wished to enroll after completing an earlier phase II trial were also eligible for EASE LID 2 enrollment but are not included in the present analyses. As in the pivotal trials, all participants were initiated at an amantadine-DR/ER dose of 137 mg/day for the first open-label trial week and titrated to 274 mg/day starting Week 2. Participants were allowed to change their PD medications (including levodopa dosage) as needed during the open-label trial, and the amantadine-DR/ER dose was tapered back to 137 mg for the final week (week 101) [15].

### 2.2 Assessments and outcomes

The primary efficacy outcome measure in the double-blind trials was change from baseline in Unified Dyskinesia Rating Scale (UDysRS) total score, as assessed at week 12 [11], [12]. Key secondary outcomes included change at 12 weeks in each patient’s PD-diary-based clinical states of OFF time, and ‘good’ ON time [defined as sum of ON time without dyskinesia and ON time with non-troublesome dyskinesia] [17]. Other secondary outcomes included changes in the MDS–UPDRS, diary-recorded ON time with troublesome dyskinesia, overall dyskinesia, and Clinician Global Impression of Change (CGI-C) [18]. All clinic-based study assessments were conducted with participants in the ON state and experiencing their typical dyskinesia. Patient home diaries were completed for the two consecutive days prior to each scheduled visit. Diaries with ≥ 4 missing entries (i.e., missing 2 h) per day were considered unevaluable for analysis. Otherwise, for each missing 30 min diary interval, data were imputed in equal portions of 15 min each, using the responses of the immediately preceding and subsequent completed (non-missing) intervals.

The MDS-UPDRS was the only PD rating instrument used in the open-label trial, with MDS-UPDRS Part IV (items 4.1–4.6) being the principal assessment of motor complications. For the present analyses we evaluated Part IV score changes from baseline in the pooled double-blind analyses through the open-label study duration.

The safety-analysis population included all patients exposed to the study drug. Safety and tolerability was assessed for the 5-2-1 cohort and was primarily based on adverse event (AE) reporting.

### 2.3 Analyses

For the purposes of these *post-hoc* analyses we evaluated the efficacy of Amantadine-DR/ER versus placebo for all phase 3 participants meeting 5-2-1 criteria at baseline who had at least one post-baseline UDysRS assessment. Double-blind data for participants who met any 2 of the 3 criteria were also assessed. Outcomes were evaluated using mixed effect model for repeated measurements (MMRM) analyses, or in the case of CGI-C, the Cochrane-Mantel-Haenszel test. All analyses were set at a two-sided, 5% significance level and were performed using SAS version 9.4 (SAS Institute Inc., Cary, North Carolina).

## 3 Results

### 3.1 Participant disposition and baseline characteristics

Of the 198 randomized participants across both double-blind trials, 105 (53.0%) used levodopa ≥ 5 times/day, 118 (59.6%) recorded ≥ 2 h of daily OFF time and 196 (99%) recorded ≥ 1-hour of daily troublesome dyskinesia. Overall, 65 patients (32.8%; n = 29 placebo; n = 36 amantadine-DR/ER) met all 3 criteria and are referred to as the ‘5-2-1 cohort’ (Fig. e1).

Participant characteristics for patients in the 5-2-1 cohort are summarized in Table 1 and details for patients meeting two of the criteria (i.e., ≥1 h of dyskinesia AND ≥ 5 doses of levodopa OR ≥ 2 h of OFF time) are presented in supplementary Table e1. On average, 5-2-1 cohort patients had been diagnosed with PD for a mean of 10.7 years and had been receiving levodopa for 8.6 years. The mean UDysRS score was 42.3 indicating severe dyskinesia, and patients were experiencing a mean of 4.1 h of OFF time per day and 4.6 h of ON Time with Troublesome Dyskinesia per day, for a total of 8.7 h (over half their waking day) spent in one of these disruptive states.

**Table 1:** Demographic and clinical characteristics for the 5-2-1 cohort at phase 3, double-blind baseline.

	Placebo (n = 28)	AMT DR/ER (n = 36)	Total (n = 64)
Age	65.3 ± 8.1	62.1 ± 9.0	63.5 ± 8.7
Male, n(%)	14 (50.0%)	23 (63.9%)	37 (57.8%)
White, n(%)	26 (92.9%)	34 (94.4%)	60 (93.8%)
Age at PD Diagnosis	55.2 ± 7.6	51.8 ± 9.5	53.3 ± 8.8
Years Since PD Diagnosis	10.6 ± 4.4	10.8 ± 5.0	10.7 ± 4.7
Duration of Levodopa treatment, years	8.6 ± 4.1	8.6 ± 4.1	8.6 ± 4.1
Duration of Dyskinesia, years	4.3 ± 2.7	4.9 ± 3.3	4.7 ± 3.1
Levodopa Daily Dose, mg	937.5 ± 586.7	1088.3 ± 517.7	1022.3 ± 549.2
LEDD, mg	1228.3 ± 605.3	1290.8 ± 579.7	1263.5 ± 587.1
UDysRS Total Score	41.0 ± 10.0	43.3 ± 12.6	42.3 ± 11.5
OFF time per day, hours	3.8 ± 1.3	4.3 ± 1.8	4.1 ± 1.6
ON with troublesome dyskinesia	4.9 ± 2.2	4.4 ± 2.1	4.6 ± 2.1
Good ON time per day, hours	7.4 ± 2.4	7.8 ± 3.2	7.7 ± 2.9
Concomitant PD medication Dopamine Agonist MAO-B Inhibitor COMT Inhibitor or Stalevo Anticholinergic	25 (89.3%) 18 (64.3%) 12 (42.9%) 10 (35.7%) 1 (3.6%)	20 (55.6%) 17 (47.2%) 10 (27.8%) 10 (27.8%) 1 (2.8%)	45 (70.3%) 35 (54.7%) 22 (34.4%) 20 (31.3%) 2 (3.1%)

All values are mean ± SD unless otherwise noted.

### 3.2 Efficacy analyses for 5-2-1 cohort

**Fig. 1:**
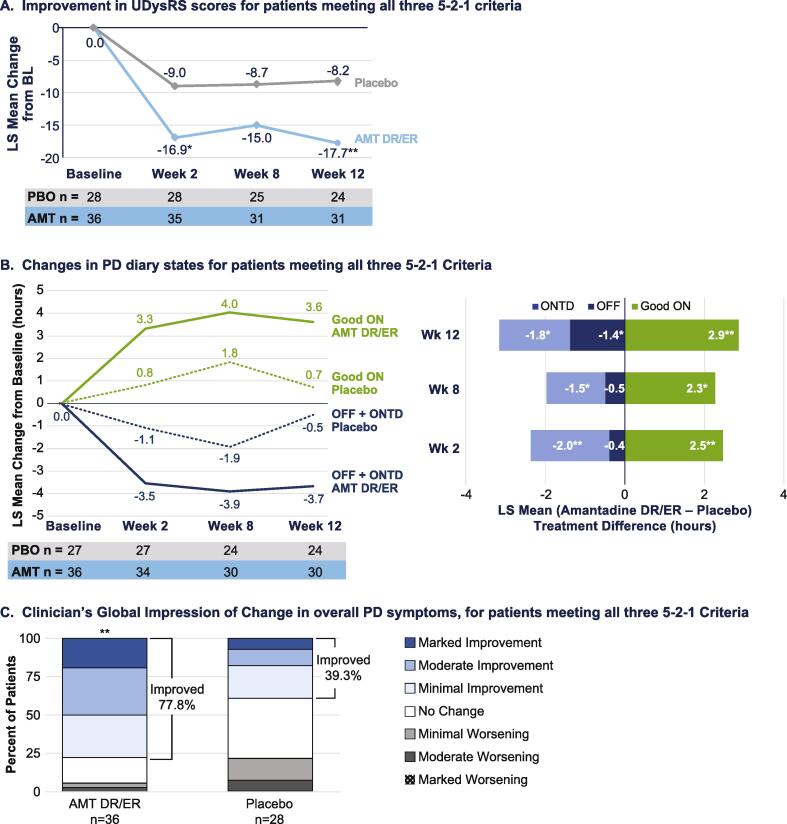
LS Mean Change from baseline in efficacy parameters during double-blind treatment for patients meeting all three 5–2-1 criteria (a) Unified Dyskinesia Rating Scale (UDysRS), (b) Changes good ON time versus OFF time + ON time with troublesome dyskinesia, based on PD diaries (c) Clinician’s Global Impression of Change (CGI-C) in overall Parkinson’s disease symptoms ratings by Treatment Group. *p < 0.05, **p < 0.01 versus placebo, analysed using MMRM for UDysRS and PD Diary measures and CMH for CGI-C. Good ON is defined as ON time without troublesome dyskinesia. AMT DR/ER: amantadine DR/ER. ONTD: ON with troublesome dyskinesia.

As shown in Fig. 1**a**, significance in dyskinesia reduction versus placebo as evidenced by UDysRS scores was seen as early as the first post baseline assessment (Week 2). At Week 12, LS mean ± SE UDysRS scores had decreased by −17.72 ± 2.20 points in the amantadine-DR/ER group compared with a −8.16 ± 2.46 point decrease in the placebo group. The treatment difference of −9.57 ± 3.15 points was statistically significant (p = 0.004) (Table 2). Based on analysis of diary states (Fig. 1**b**), treatment with amantadine DR/ER improved good ON time (ON time without Troublesome Dyskinesia), with significant differences also emerging at Week 2. At Week 12, LS mean ± SE good ON time had increased by 3.6 ± 0.6 h in the amantadine-DR/ER group compared with an increase of 0.7 ± 0.7 h in the placebo group; the LS mean ± SD treatment difference was 2.9 ± 0.90 h/day and was statistically significant (p = 0.002). Reductions in OFF time and ON with troublesome dyskinesia mirrored the improvements in good ON time, indicating that the benefits in improving good ON time were driven by reductions in both OFF time and ON time with troublesome dyskinesia.

**Table 2:** Treatment differences for amantadine-DR/ER versus placebo for patients meeting all three 5-2-1 criteria.

	Placebo	Amantadine-DR/ER
**UDysRS**		
**Week 2** Change from baseline Treatment effect vs placebo P value	−8.95 ± 2.39; n = 28	−16.92 ± 2.17; n = 35 −7.95 ± 3.08 0.012
**Week 8** Change from baseline Treatment effect vs placebo P value	−8.72 ± 2.57; n = 25	−14.97 ± 2.33; n = 31 −6.24 ± 3.32 0.066
**Week 12** Change from baseline Treatment effect vs placebo P value	−8.16 ± 2.46; n = 24	−17.73 ± 2.20; n = 31 −9.57 ± 3.15 0.004

**Good ON time (hours)**		
**Week 2** Change from baseline Treatment effect vs placebo P value	0.82 ± 0.62; n = 27	3.30 ± 0.56; n = 34 2.48 ± 0.78 0.002
**Week 8** Change from baseline Treatment effect vs placebo P value	1.76 ± 0.71; n = 24	4.01 ± 0.64; n = 30 2.25 ± 0.91 0.02
**Week 12** Change from baseline Treatment effect vs placebo P value	0.69 ± 0.70; n = 24	3.59 ± 0.63; n = 30 2.90 ± 0.90 0.002

**OFF time (hours)**		
**Week 2** Change from baseline Treatment effect vs placebo P value	−0.17 ± 0.35; n = 27	−0.59 ± 0.31; n = 34 −0.42 ± 0.44 0.35
**Week 8** Change from baseline Treatment effect vs placebo P value	−0.02 ± 0.42; n = 24	−0.54 ± 0.38; n = 30 −0.52 ± 0.55 0.35
**Week 12** Change from baseline Treatment effect vs placebo P value	0.70 ± 0.45; n = 24	−0.72 ± 0.40; n = 30 −1.42 ± 0.58 0.02

**ON time with troublesome dyskinesia (hours)**		
**Week 2** Change from baseline Treatment effect vs placebo P value	−0.96 ± 0.53; n = 27	−2.95 ± 0.47; n = 34 −1.99 ± 0.67 0.004
**Week 8** Change from baseline Treatment effect vs placebo P value	−1.84 ± 0.56; n = 24	−3.36 ± 0.50; n = 30 −1.52 ± 0.70 0.035
**Week 12** Change from baseline Treatment effect vs placebo P value	−1.17 ± 0.60; n = 24	−2.96 ± 0.54; n = 30 −1.78 ± 0.77 0.024

**MDS-UPDRS Part II (ADL)**		
**Week 2** Change from baseline Treatment effect vs placebo P value	−2.24 ± 0.99; n = 24	−4.17 ± 0.86, n = 34 −1.93 ± 1.30 0.14
**Week 8** Change from baseline Treatment effect vs placebo P value	−2.01 ± 0.80; n = 25	−3.40 ± 0.73; n = 31 −1.39 ± 1.03 0.18
**Week 12** Change from baseline Treatment effect vs placebo P value	−1.35 ± 0.80; n = 24	−4.10 ± 0.72; n = 31 −2.74 ± 1.02 0.009

**MDS-UPDRS Part IV (motor complications)**		
**Week 2** Change from baseline Treatment effect vs placebo P value	−1.52 ± 0.53; n = 27	−3.94 ± 0.48; n = 34 −2.43 ± 0.69 0.001
**Week 8** Change from baseline Treatment effect vs placebo P value	−2.26 ± 0.58; n = 25	−3.91 ± 0.52; n = 31 −1.65 ± 0.75 0.031
**Week 12** Change from baseline Treatment effect vs placebo P value	−1.80 ± 0.53; n = 24	−3.56 ± 0.47; n = 31 −1.77 ± 0.67 0.011

All values are LS mean ± SE from the MMRM model with change from baseline as the dependent variable and the baseline value as a covariate. The model includes categorical effects for treatment group, study, and visit (Weeks 2, 8, and 12), and the interaction between treatment group and visit. Good ON time is defined as ON time without troublesome dyskinesia.

The clinical relevance of these dyskinesia and OFF time reductions is supported by significant improvement in CGI (*P* = 0.002) versus placebo. At Week 12, 77.8% of patients treated with amantadine-DR/ER reported improvement versus 39.3% with placebo (Fig. 1**c**). Significant improvements were also noted for amantadine-DR/ER versus placebo for UPDRS Part II (activities of daily living) and Part IV (complications of therapy) (Table 2). Treatment with amantadine-DR/ER was similarly effective across all outcome measures throughout the double-blind studies for patients meeting two out of three 5-2-1 criteria (Table e2).

During open-label treatment, mean MDS-UPDRS Part IV scores assessing presence and severity of motor complications were maintained below double-blind baseline levels, throughout open-label treatment to Week 100 (Fig. e2).

### 3.3 Safety and tolerability analyses

Table 3 summarizes adverse event reporting for the 5-2-1 cohort as well as data previously reported for the overall phase 3 trials safety cohort [13]. Consistent with the overall safety cohort most AEs reported in patients meeting 5-2-1 criteria were of mild to moderate intensity. For 5-2-1 cohort patients treated with amantadine DR/ER, the most common events were falls, peripheral edema, dizziness, and hallucinations (primarily visual). Serious AEs were reported for 8.3% of 5-2-1 patients treated with amantadine-DR/ER versus 6.9% treated with placebo, no serious AE was considered related to study drug.

**Table 3:** Subjects [n (%)] experiencing adverse events in the 5–2-1 cohort versus all patients receiving amantadine-DR/ER in the double-blind, phase 3 trials [13].

	5–2-1 Cohort (All 3 criteria)^c^		Pooled Phase 3 [13]
	Placebo (n = 29)	AMT DR/ER (n = 36)	AMT DR/ER (n = 100)
Any AE	12 (41.4%)	30 (83.3%)	87.0%
Study-drug related AEs	4 (13.8%)	20 (55.6%)	61.0%
Serious AEs	2 (6.9%)	3 (8.3%)	11.0%
Discontinued due to AEs	2 (6.9%)	7 (19.4%)	20.0%

**Most frequent AEs (>2 patients in any 5**–**2-1 cohort group)**			
Fall	3 (10.3%)	8 (22.2%)	13.0%
Peripheral Edema	0	8 (22.2%)	15.0%
Dizziness	0	7 (19.4%)	16.0%
Dry Mouth	0	6 (16.7%)	16.0%
Hallucinations (all types)	1 (3.4%)	6 (16.7%)	21.0%
Constipation	2 (6.9%)	4 (11.1%)	13.0%
Nausea	1 (3.4%)	4 (11.1%)	8.0%
Anxiety	1 (3.4%)	3 (8.3%)	7.0%
Back Pain	0	3 (8.3%)	4.0%
Contusion	1 (3.4%)	3 (8.3%)	6.0%
Insomnia	0	3 (8.3%)	6.0%
Urinary Tract Infection	2 (6.9%)	3 (8.3%)	8.0%

Safety and tolerability during the open-label follow-on study was similar, with half (51.1%) of patients reporting a treatment-related AE. The most common treatment-related AEs with long-term treatment were hallucinations (22.2%) and peripheral edema (13.3%); 6.7% of patients reported falls that were assessed as treatment-related. Overall, 31.1% of patients reported serious AEs (6.7% related to study drug) and 22.2% of 5-2-1 patients discontinued treatment with amantadine-DR/ER due to AEs.

## 4 Discussion

In this analysis of patients meeting 5-2-1 criteria, treatment with amantadine-DR/ER reduced dyskinesia severity as assessed by UDysRS scores and resulted in an increase in good ON time of 3.6 h versus baseline and 2.9 h versus placebo. Improvements in good ON time resulted from significant reductions in both troublesome dyskinesia and OFF time with AEs consistent with those known to occur in patients with advanced PD and with amantadine.

As PD progresses, the so called ‘therapeutic window’ for levodopa narrows and maintaining a consistent symptomatic benefit while controlling dyskinesia becomes increasingly difficult. At this stage, the addition of oral adjunct treatments for OFF motor fluctuations in PD can result in or exacerbate dyskinesia. The 5-2-1 criteria were proposed to identify patients with PD who are poorly controlled on oral therapy and may benefit from device-assisted therapies such as continuous subcutaneous apomorphine or levodopa infusion, or DBS [2]. A basic assumption for 5-2-1 criteria is that patients have had their oral dopaminergic treatment regimens ‘optimized’, and yet are still poorly controlled. However, there is no single definition of what ‘optimized’ refers to and our findings suggest that a sizeable proportion of patients who might be considered good candidates for DAT could potentially benefit from amantadine-DR/ER. Amantadine-DR/ER is the only medication FDA-approved for the treatment of both dyskinesia and OFF episodes in levodopa-treated patients with PD. Our findings would suggest that, if a patient hasn’t already tried amantadine-DR/ER, it should be considered in appropriate patients before moving on to DAT.

In their recent analysis of an ongoing observational study, Aldred et al. [19] found that only 20% of patients being treated with LCIG met all three 5-2-1 criteria at initiation of therapy but 68% of patients met two of the three criteria, suggesting that a significant proportion of patients are referred for invasive DAT before they meet the expert-recommended criteria [2]. In our analyses, the efficacy of amantadine-DR/ER for 5-2-1 patients was in line with the overall phase-3 studies [11], [12], [13] highlighting the consistent efficacy of amantadine-DR/ER, even in this more severely affected subgroup. For patients who met just two of the three criteria, treatment with amantadine-DR/ER was also consistently effective across the different outcome measures, with a slightly larger magnitude of effect than seen in the more affected 5-2-1 population. Of note, even for the more advanced 5-2-1 population, efficacy was maintained throughout 100 weeks of open-label amantadine-DR/ER treatment, suggesting the benefit in treating motor complications persists. By the end of the 100-week follow-up, motor fluctuation scores remained below baseline levels.

The safety profile of amantadine-DR/ER for patients in this more severely affected 5-2-1 group was generally in line with that previously reported for the overall population [13]. However, we do note that the incidence of falls in the 5-2-1 group (22.2%) was higher than was observed in the overall AMT DR/ER group. This could potentially be related to more advanced disease state and/or more OFF time since rates of falls were also higher in the placebo-treated group. Although we cannot compare the safety of amantadine-DR/ER with what *would* have happened if these 5-2-1 patients had started on DAT, orally-administered amantadine-DR/ER is not associated with the significant surgical risks associated with LCIG or DBS [20], [21], nor the local skin reactions associated with apomorphine infusion [22].

Strengths of this analysis include the similar design and conduct of the two pivotal trials which allowed pooling of the data to enable robust analysis of the 5-2-1 subgroup. However, we acknowledge several limitations, including the relatively small size and *post-hoc* nature of the 5-2-1 subgroup analyses and the open-label nature of the follow-on trial. Whereas patients in the study reported by Aldred primarily met levodopa dose and OFF time criteria [19], patients in the EASE-LID studies were recruited based on dyskinesia which might imply differences in patient populations when the criteria are applied.

In conclusion, as PD progresses, treatment with oral therapies becomes increasingly challenging. Many patients have poor motor control despite multiple daily doses of levodopa and use of adjunctive dopaminergic therapies. While these patients may benefit from DAT, these therapies are associated with their own set of burdens and risks, and not all patients with advanced disease are good candidates [23]. It is therefore important to consider the potential benefits of amantadine-DR/ER in appropriately selected patients.

## 5 Data sharing statement

Where patient data can be anonymized, Adamas Pharmaceuticals Inc will share all individual participant data that underlie the results reported in this article with qualified researchers who provide valid research question(s). Study documents, such as the study protocol and clinical study report, are not always available.

## Author contributions

All authors contributed to the analysis plan, interpretation of results and approved the final version of the article. RAH conceived the project, AEF directed the data analysis and RAH and AEF wrote the first draft of the manuscript. RAH was an investigator in the ADS-5102 pivotal studies. SG participated in the project design and provided critical review of the draft.

## Funding

This work was funded by Adamas Pharmaceuticals, Inc.

## Declaration of Competing Interest

The authors declare the following financial interests/personal relationships which may be considered as potential competing interests: Robert A Hauser is supported in part by a Center of Excellence grant from the National Parkinson Foundation and is employed by the University of South Florida (Florida). He received payment from Adamas Pharmaceuticals, Inc. for participating as a Steering Committee member and reports receiving personal fees from Abbvie, Acorda, Adamas, Alterity, Amneal, Cerevance, Curium Pharma, Enterin, Global Kinetics Corp., Inhibikase, Jazz Pharmaceuticals, Kyowa Kirin, Merck, Merz, Neurocrine, NeuroDerm, Orion, Pharmather Inc., Sage Therapeutics, Scion, Sio Gene Therapies, Sunovion, Supernus, Tolmar, and Vivifi Biotech. Santosh Goud and Andrea E. Formella are employed by and own stock in Adamas Pharmaceuticals Inc.
