# Supplementary material for: Potential utility of amantadine DR/ER in persons with Parkinson’s disease meeting 5-2-1 criteria for device aided therapy
Source: Clin Park Relat Disord. 2021 Dec 8;6:100123. doi: 10.1016/j.prdoa.2021.100123 (PMC8760552; doi:10.1016/j.prdoa.2021.100123)
Supplement: Supplementary data 1 [file mmc1.pdf]

## Supplemental Appendix

**Figure e1**. Disposition of patients meeting all three 5-2-1 criteria across amantadine-DR/ER phase 3, double-blind* and open-label studies.

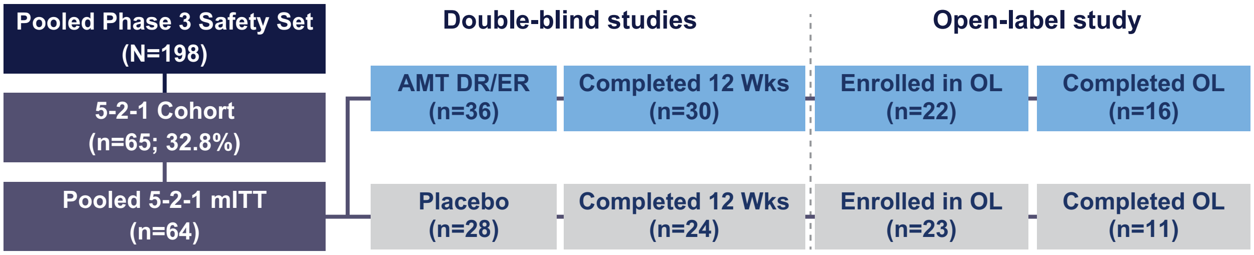


**Pooled studies: EASE LID was a 25-week study and EASE LID 3 was a 13-week study.*

**n=30 (47% of 5-2-1 cohort completed open-label week 100: n=27 completed the trial (n=16 from amantadine DR/ER group and n=11 from former placebo group). A total of 30 patients (47% of 5-2-1 cohort) completed open-label week 100. Three patients dropped out prior to completing the end of study medication taper.*

**Figure e2 Mean change from double-blind baseline in MDS-UPDRS Part IV motor complications scores through the open-label follow-on study for patients meeting all three 5-2-1 criteria**

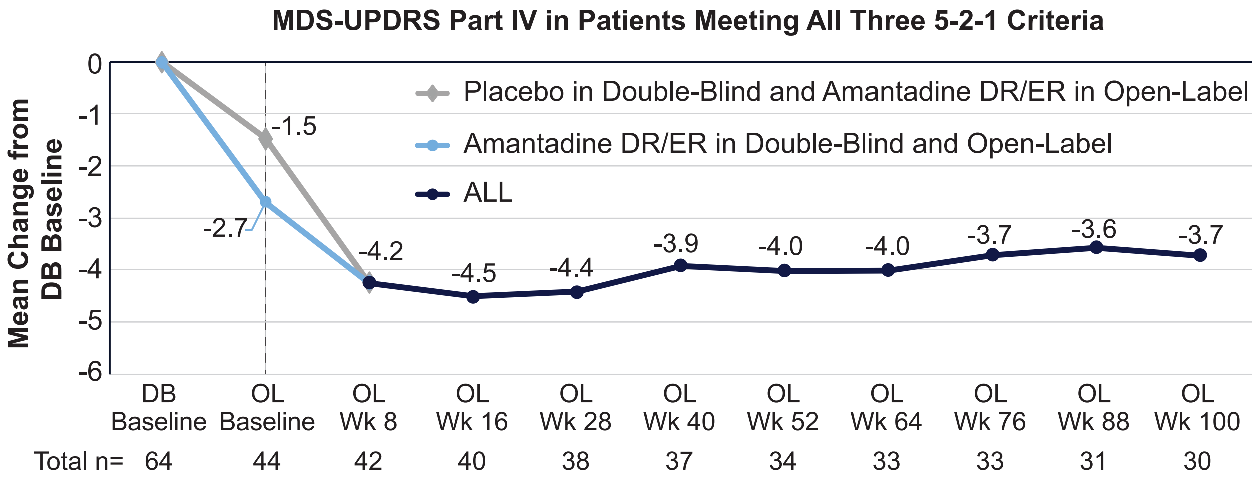

**Table e1.** Baseline demographic and clinical characteristics for patients meeting two of the three 5-2-1 criteria

| | **Placebo**<br><br>**N=75** | **Amantadine-DR/ER**<br><br>**N=80** |
|---|---|---|
| Age, years | 64.5 ± 8.8 | 62.7 ± 9.7 |
| Male, n(%) | 46 (61.3%) | 45 (56.3%) |
| White, n(%) | 69 (92.0%) | 77 (96.3%) |
| Age at PD Diagnosis | 55.2 ± 8.4 | 53.0 ± 9.7 |
| Years Since PD Diagnosis | 9.9 ± 3.9 | 10.2 ± 4.7 |
| Duration of Levodopa treatment, years | 7.9 ± 4.1 | 8.3 ± 3.9 |
| Duration of Dyskinesia, years | 3.7 ± 2.6 | 4.3 ± 3.2 |
| Levodopa Daily Dose, mg | 771.6 ± 477.0 | 894.5 ± 504.7 |
| LEDD, mg | 1043.4 ± 495.8 | 1128.8 ± 571.3 |
| UDysRS Total Score | 39.8 ± 11.0 | 42.1 ± 12.9 |
| OFF time per day, hours | 3.1 ± 1.9 | 3.5 ± 2.1 |
| ON with troublesome dyskinesia | 5.2 ± 2.8 | 4.8 ± 2.4 |
| Good ON time per day, hours | 7.7 ± 2.8 | 8.0 ± 3.0 |
| Concomitant PD medication | | |
| Dopamine Agonist | 45 (60.0%) | 40 (50.0%) |
| MAO-B Inhibitor | 35 (46.7%) | 33 (41.3%) |
| COMT Inhibitor or Stalevo | 31 (41.3%) | 28 (35.0%) |
| Anticholinergic | 4 (5.3%) | 2 (2.5%) |

*All values are mean ± SD unless otherwise noted*

**Table e2**. Treatment differences for DR-ER amantadine versus placebo for patients meeting and two of the three 5-2-1 criteria

| | Amantadine-DR/ER vs. Placebo | P value |
|---|---|---|
| UDysRS, LS mean ± SE | | |
| Week 2 | -11.53 ± 1.86 | <0.0001 |
| Week 8 | -9.81 ± 2.15 | <0.0001 |
| Week 12 | -11.30 ± 2.08 | <0.0001 |
| Good ON time (hours), LS mean ± SE | | |
| Week 2 | 2.53 ± 0.47 | <0.0001 |
| Week 8 | 2.59 ± 0.58 | <0.0001 |
| Week 12 | 2.57 ± 0.53 | <0.0001 |
| OFF time (hours), LS mean ± SE | | |
| Week 2 | -0.44 ± 0.27 | 0.1067 |
| Week 8 | -0.77 ± 0.31 | 0.0147 |
| Week 12 | -1.18 ± 0.34 | 0.0007 |
| ON time with troublesome dyskinesia (hours), LS mean ± SE | | |
| Week 2 | -1.94 ± 0.40 | <0.0001 |
| Week 8 | -1.59 ± 0.42 | 0.0002 |
| Week 12 | -1.60 ± 0.45 | 0.0005 |
| MDS-UPDRS Part II (ADL), LS mean ± SE | | |
| Week 2 | -1.93 ± 0.77 | 0.0126 |
| Week 8 | -1.58 ± 0.73 | 0.0311 |
| Week 12 | -2.29 ± 0.73 | 0.0021 |
| MDS-UPDRS Part IV (motor complications), LS mean ± SE | | |
| Week 2 | -2.72 ± 0.46 | <0.0001 |
| Week 8 | -1.88 ± 0.51 | 0.0003 |
| Week 12 | -2.74 ± 0.47 | <0.0001 |

**Table e3**. Adverse events reported for patients meeting any two of the three 5-2-1 criteria

| | **Patients meeting any 2 of the 3 criteria for device aided therapies** | | |
|---|---|---|---|
| | Placebo (n=76) | AMT DR/ER (n=80) | Total (n=156) |
| Any AE | 41 (53.9%) | 69 (86.3%) | 70.5% |
| Study drug related AEs | 11 (14.5%) | 47 (58.8%) | 37.2% |
| Serious AEs | 3 (3.9%) | 10 (12.5%) | 8.3% |
| % Subjects discontinued due to AEs | 4 (5.3%) | 14 (17.5%) | 11.5% |
| **Most common AEs** | | | |
| Hallucinations (Pooled) | 2 (2.6%) | 15 (18.8%) | 10.9% |
| Peripheral Edema | 1 (1.3%) | 13 (16.3%) | 9.0% |
| Dizziness | 1 (1.3%) | 13 (16.3%) | 9.0% |
| Dry Mouth | 0 | 13 (16.3%) | 8.3% |
| Fall | 6 (7.9%) | 11 (13.8%) | 10.9% |
| Constipation | 3 (3.9%) | 10 (12.5%) | 8.3% |
| Nausea | 2 (2.6%) | 8 (10.0%) | 6.4% |
